# Comparative efficacy of ravulizumab and eculizumab in the treatment of atypical hemolytic uremic syndrome: An indirect comparison using clinical trial data 

**DOI:** 10.5414/CN110516

**Published:** 2021-12-21

**Authors:** Ioannis Tomazos, Anthony J. Hatswell, Spero Cataland, Peter Chen, Nick Freemantle, Åsa Lommele, Kevin Deighton, Emma Knowles, Neil S. Sheerin, Eric Rondeau

**Affiliations:** 1 Alexion, AstraZeneca Rare Disease, Boston, MA,; 2 Delta Hat Limited, Nottingham, UK,; 3 Ohio State University, Columbus, OH, USA,; 4 University College London, London,; 5 Newcastle University, Newcastle, UK,; 6 Hôpital Tenon, APHP and Sorbonne Université, Paris, France

**Keywords:** acute kidney injury, chronic kidney disease, propensity scoring, stabilized weights, terminal complement inhibition

## Abstract

Ravulizumab and eculizumab are approved terminal complement inhibitor treatments for atypical hemolytic uremic syndrome (aHUS). Ravulizumab was engineered from eculizumab to have an increased half-life allowing for reduced dosing frequency (8-weekly vs. 2-weekly). To account for differences in respective clinical trials, a validated balancing technique was used to enable an indirect comparison of ravulizumab and eculizumab treatment efficacy in aHUS. Patient-level data from four eculizumab clinical trials were available for pooling and comparison with data from two ravulizumab trials. In the primary analysis, adult native kidney data were compared. Propensity scores were calculated from baseline characteristics (dialysis status, estimated glomerular filtration rate, platelet count, serum lactate dehydrogenase). Stabilized inverse probability weighting was used to balance groups. Changes in outcomes from baseline to 26 weeks were compared between treatment groups. Sensitivity and subgroup analyses were conducted to assess the robustness of findings. Overall, 85 patients (46 ravulizumab, 39 eculizumab) were included in the primary analysis. Demographic and clinical characteristics were well balanced after weighting at baseline. At 26 weeks, clinical outcomes (including renal function, hematological markers, and dialysis prevalence), and fatigue and quality of life measures were improved with eculizumab and ravulizumab treatment. No differences between treatment groups reached statistical significance, although confidence intervals were wide. Sensitivity and subgroup analysis results were consistent with those of the primary analysis. Using appropriate methodology for indirect comparison of studies, no differences in outcomes were seen between ravulizumab and eculizumab, although, owing to small sample sizes, confidence intervals were wide.

## Introduction 

Atypical hemolytic uremic syndrome (aHUS) is a rare disease caused by uncontrolled terminal complement activation that can lead to severe, progressive organ damage or death if left untreated [1]. The 2011 US and EU approvals of eculizumab, a humanized monoclonal antibody that blocks terminal complement activation at C5, brought about a paradigm shift in the management of aHUS with eculizumab becoming the standard of care [2, 3]. Previous treatment and disease management options (plasma exchange/infusion, dialysis for renal failure, and kidney transplantation) were limited, and associated outcomes were poor [4, 5, 6, 7]. Over the past 10 years, the efficacy and safety of eculizumab have been demonstrated in both adult and pediatric prospective clinical trials [8, 9, 10, 11, 12], with additional data from registry and real-world patient studies [7, 13, 14, 15]. Although highly effective at treating patients with aHUS, eculizumab must be administered every 2 weeks in patients weighing 10 kg or more [2, 3]. 

Ravulizumab, a rapid and long-acting C5 inhibitor, was engineered from eculizumab to achieve extended complement inhibition while retaining the clinical benefits and favorable safety profile of eculizumab [16]. The efficacy and safety of ravulizumab have been evaluated in single-arm clinical trials in adults [17] and in pediatric/adolescent patients [18, 19]. The increased half-life of ravulizumab due to antibody recycling allows for a reduced dosing frequency compared with eculizumab (8-week infusion intervals; 4 weeks for patients under 20 kg) [20, 21], thus reducing treatment burden while continuing the preservation of renal function and decreasing the need for dialysis. 

A head-to-head comparison of ravulizumab and eculizumab found that ravulizumab given every 8 weeks achieved noninferiority compared with eculizumab given every 2 weeks for all efficacy endpoints assessed in complement inhibitor-naive adults with paroxysmal nocturnal hemoglobinuria (PNH), a disease for which both ravulizumab and eculizumab are also indicated [22]. PNH has a clear diagnostic algorithm and an estimated prevalence that is ~ 6 times higher than that for aHUS, making head-to-head comparison feasible [23, 24, 25]. The low prevalence of aHUS and heterogeneity in disease presentation does not allow for such a head-to-head comparison, while a naive comparison of data from the respective clinical studies is likely to be biased owing to differences in the patient populations enrolled, study designs, and endpoint definitions. In order to present statistically unbiased estimates of comparative effectiveness, indirect comparison methods are therefore required. This study uses propensity scoring techniques to illustrate the effectiveness of ravulizumab and eculizumab for the treatment of aHUS, accounting for observed differences between clinical studies. 

## Materials and methods 

### Patient population 

Complement inhibitor-naive patient-level data from eculizumab clinical trials C08-002A/B (NCT00844545/NCT00844844), C08-003A/B (NCT00838513/NCT00844428), C10-003 (NCT01193348), and C10-004 (NCT01194973) were available for pooling and comparison with data from ravulizumab trials ALXN-aHUS-311 (NCT02949128) and ALXN-aHUS-312 (NCT03131219). Data from eculizumab trial C08-003 were excluded owing to the study being conducted in a different patient population (patients were receiving long-term maintenance plasma therapy at baseline and, consequently, had normal platelet counts) [9]. Data from eculizumab trial C11-003 (NCT01522170) were excluded because this is the long-term follow-up study of patients with aHUS who were treated with at least one infusion of eculizumab in any of five previously conducted parent studies (C08-002, C08-003, C10-003, C10-004, and C09-001r (retrospective, observational)), with baseline being the end of the parent trial. 

Kidney transplant recipients and pediatric patients (aged ≤ 18 years) were excluded from the primary analysis to ensure a homogeneous group for comparison; thus trials C08-002B and ALXN-aHUS-312 were excluded in the primary analysis because only pediatric patients were enrolled. Kidney transplant recipients and pediatric patients were examined in subgroup analyses. Adult patients with native kidneys represented the largest population group within both the ravulizumab and eculizumab trial data sets and thus constituted the primary analysis (study designs for eculizumab trials C08-002A and C10-004, and ravulizumab trial ALXN-aHUS-311 have been described previously [9, 11, 17]). For inclusion in the primary analysis, all patients were required to have complete baseline data for the variables used within the propensity score specification (dialysis status, estimated glomerular filtration rate (eGFR), platelet count, and serum lactate dehydrogenase (LDH) concentration) to allow for the balancing of baseline patient characteristics between treatments. Patients were permitted to have a maximum of one missing laboratory variable at baseline or endpoint for serum creatinine (SCr) concentration (for non-dialysis patients), eGFR, LDH concentration, and platelet count. Death was considered as an outcome, thus patients who died were also included in the analysis despite clinical laboratory measures not being available. 

### Outcomes data 

Data collected at 26 weeks were used for outcomes analysis. If a patient had more than one visit within the “window of acceptability” (± 56 days), the observation taken on the day closest to day 183 (26 weeks) was adopted as the endpoint to ensure the maximum inclusion of patient data and to allow an informed, robust comparison between treatments. 

Definitions for clinical outcomes (SCr concentration improvement, LDH normalization, platelet count normalization, and complete thrombotic microangiopathy (cTMA) response) were aligned with the ravulizumab clinical study for consistency, including having eGFR set to a value of 10 mL/min/1.73m^2^ for patients undergoing dialysis [17]. In accordance with clinical guidance, SCr values were included only for patients not undergoing dialysis to ensure the validity of the measure. Although there were differences between study protocols in how dialysis status was defined, these were aligned as closely as possible at both baseline and endpoint. Table 1 shows the aligned definitions used for dialysis status and clinical outcome measures. 

### Statistical analyses 

**Propensity scoring**

Propensity scoring was performed to balance the treatment groups according to observed baseline characteristics, thus providing a basis for comparison of eculizumab and ravulizumab [26]. Propensity scores were calculated at baseline for each patient in the primary analysis using dialysis status, eGFR, platelet count, and serum LDH (listed in hierarchical order of importance). The propensity scoring method for balancing prognostic baseline characteristics between treatments used the following formula after refactoring the data for LDH into upper and lower halves to ensure consistent overlap between treatment groups across all propensity score values: 

Treatment ~ dialysis + eGFR + platelets + LDH (halves) 

**Stabilized weights**

The resulting propensity scores were then used to balance baseline patient characteristics between treatment groups via the implementation of stabilized inverse propensity score weights. The application of stabilized weights preserves the sample sizes in the analysis data set and allows for appropriate estimates of the variance to be given without complex methodology [27]. Stabilized weight, *SW*, assigned for each individual, *i*, based on propensity score,* π*, and the proportion of treated patients, p, is given as: [Fig Equation1]

The effective sample size is calculated as the sum of outcome weights in each treatment group. Statistical tests were performed after application of the stabilized weights to ensure that prognostic baseline characteristics were not significantly different at the p < 0.1 level before endpoints were revealed. 

### Treatment group comparison 

Welch’s two-way t-tests were used for all continuous variables while χ^2^-tests were used to obtain p-values for categorical variables. Owing to the limitations of the χ^2^-test in terms of sample size, p-values are presented only when five or more observations were present for each category. Values between treatments at endpoint were assessed for differences at a significance level of 0.05. Confidence intervals (CIs) of the difference between ravulizumab and eculizumab were provided for all continuous variables. CIs of the difference in proportions were provided for categorical variables with binary outcomes. 

### Sensitivity and subgroup analyses 

In a sensitivity analysis of the propensity scoring approach used, propensity score matching (as opposed to the weighting used in the primary analysis) was performed using the procedure of 1 : 1 matching (each patient in the “treated” (ravulizumab) group was matched with the control (eculizumab) patient exhibiting the nearest propensity score). A caliper width of 0.2 times the standard deviation of the propensity score was used [28] and “random” order for matching. 

To test the robustness of findings from the primary analysis, sensitivity analyses were conducted whereby the window of acceptability for data collection was reduced to 28 days, patients resident in Asian countries were excluded, patients who died during the trial period were excluded, and patients aged at least 65 years were excluded. 

In addition to sensitivity analyses, separate subgroup analyses were conducted using alternative patient populations – adults with prior kidney transplant and pediatric native kidney patients. Patients included in these analyses were required to have data availability in line with the primary analysis. Pediatric patients with prior kidney transplant were not evaluated owing to low patient numbers. 

### Statistical software 

All analyses were performed using the statistical software R version 3.6.3 [29] implemented with the package MatchIt [30]. 

## Results 

### Primary analysis 

**Figure 1:**
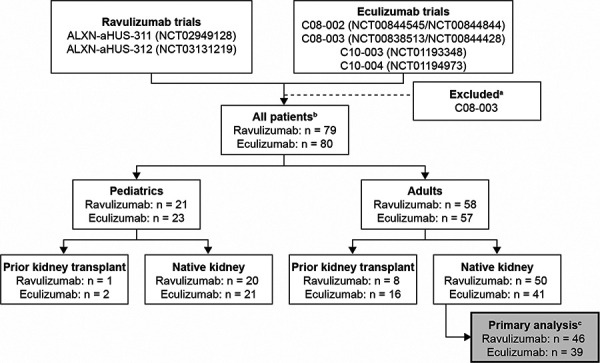
Flow of patients included in the primary analysis dataset. ^a^Data from eculizumab trial C08-003 were excluded owing to the study being conducted in a different patient population (patients were receiving long-term maintenance plasma therapy at baseline and, consequently, had normal platelet counts). ^b^“All patients” represents the maximum possible number of patients before the application of missing data restrictions for each subgroup by treatment. ^c^Patient numbers for primary analysis represent patients with complete cases for propensity score variables, with a maximum of one missing laboratory measure and outcome data within 56 days of the 6-month endpoint.

In total, 85 complement inhibitor-naive adult native kidney patients were included in the primary analysis (patient flow is shown in Figure 1). Of these, 46 received ravulizumab 10 mg/mL, and 39 received eculizumab 10 mg/mL in their respective trials. 

Patient characteristics at baseline, with application of stabilized weights, are shown in Table 2. Baseline characteristics were generally well balanced between eculizumab and ravulizumab groups with no clear discrepancies in any patient characteristics or quality of life and fatigue measures. These include the proportion of patients undergoing dialysis, mean platelet count, and eGFR. Although patients in the ravulizumab cohort were older than those in the eculizumab cohort (mean (standard deviation) age: 40 (14) vs. 34 (13) years, respectively. 95% CI: 0 – 12; p = 0.050), there was no difference in the proportion of older adults aged at least 65 years between treatment groups, therefore the difference in mean age was not thought to be clinically relevant. 

With the application of stabilized weights, outcomes at 26 weeks were improved from baseline in both the eculizumab and ravulizumab groups (Table 3). These included the proportion of patients undergoing dialysis, SCr concentration, eGFR, platelet count, and serum LDH concentration. Improvements were also seen with both treatments in terms of quality of life and fatigue, as measured using the 5-dimension EuroQol questionnaire (EQ-5D) and the Functional Assessment of Chronic Illness Therapy-Fatigue (FACIT-F) instruments, respectively. 

No consistent pattern was seen between eculizumab and ravulizumab groups at 26 weeks in hematological and renal outcome point estimates (Table 3). No differences in outcomes between treatment groups reached statistical significance, although the wide confidence intervals should be noted. The proportion of patients undergoing dialysis at 26 weeks was numerically better for eculizumab than for ravulizumab (8% (95% CI: 3 – 21%) vs. 22% (95% CI: 13 – 37%), respectively), with overall renal function (as measured by eGFR) numerically in favor of ravulizumab (mean (standard deviation): 55.4 (40.8) mL/min/1.73m^2^ vs. 51.4 (30.8) mL/min/1.73m^2^ for eculizumab). Three deaths were reported in the ravulizumab group, and no deaths in the eculizumab group. The proportion of patients in both treatment groups achieving improvement/normalization in key clinical parameters ranged from 59 to 96%, with cTMA response achieved in 70% (95% CI: 54 – 82%) and 61% (95% CI: 46 – 74%) of eculizumab and ravulizumab patients, respectively (Table 4). 

### Sensitivity analyses 

When applying the alternative approach of propensity score matching, 29 patients in each treatment group were matched; results were consistent with the primary analysis and are presented in full in Supplemental Table 1. Using alternative specifications of the primary analysis population, all sensitivity analyses (reduced window of acceptability, exclusion of patients resident in Asian countries, exclusion of deaths and exclusion of patients aged ≥ 65 years) showed substantial improvement in outcomes for both eculizumab and ravulizumab and an absence of separation between the treatment groups, consistent with the primary analysis (data not shown). However, it should be noted that there remains uncertainty in the relative effects owing to patient numbers being reduced compared with the primary analysis population in many cases (Supplemental Table 2). 

### Subgroup analyses 

Additional subgroups of patients with aHUS from the original trials were also evaluated, specifically adults with prior kidney transplant and pediatric native kidney patients. Baseline characteristics for adults with prior kidney transplant (eculizumab, n = 15; ravulizumab, n = 7) and pediatric native kidney patients (eculizumab, n = 20; ravulizumab, n = 12) are shown in Supplemental Tables 3 and 4. At 26 weeks, these populations demonstrated a notable improvement compared with baseline following eculizumab and ravulizumab treatment (Supplemental Tables 5 and 6). In adults with prior kidney transplant, cTMA response was achieved in 59% (95% CI: 32%, 81%) and 82% (95% CI: 49%, 96%) of eculizumab and ravulizumab patients, respectively, and 67% (95% CI: 46%, 83%) and 77% (95% CI: 47%, 93%) of patients in the pediatric native kidney subgroup. Interpretation of the relative treatment effects is limited by the number of patients included in the analyses. 

## Discussion 

In the absence of a head-to-head clinical trial, this study provides a formal estimate of the efficacy of ravulizumab and eculizumab for the treatment of patients with aHUS. Results of the analysis show that treatment with ravulizumab or eculizumab leads to substantial improvements in clinical outcomes including renal function, hematological markers, and dialysis prevalence, as well as improvements in measures of fatigue and quality of life [9, 11, 17]. Better outcomes in patient-relevant measures such as fatigue or quality of life should ultimately mean that patients are not only able to lead a more robust lifestyle but also have a greater likelihood of improved performance at work/school. After aligning endpoint definitions between trials and adjusting for the baseline characteristics of study populations using propensity score weighting/matching techniques (along with multiple sensitivity analyses), point estimates of outcomes appear similar, although the confidence intervals around these point estimates of differences were wide, owing to the limited number of patients included in the clinical studies. 

After the application of stabilized weights, there were some instances of numerical differences between ravulizumab and eculizumab in treatment outcomes, although none reached statistical significance. For example, the proportion of patients undergoing dialysis at week 26 was higher with ravulizumab than with eculizumab, while renal function (as measured by eGFR at 26 weeks in all patients) was better with ravulizumab than with eculizumab. There was also a numerical difference in the number of deaths recorded, with three deaths in the ravulizumab cohort and no deaths in the eculizumab cohort. None of these deaths were deemed to be treatment-related by the original investigators of the clinical trials [17]. 

A recent meta-analysis and pairwise comparison of eculizumab and ravulizumab clinical trials and real-life studies in aHUS found no apparent difference between the effectiveness of the two treatments [31]. Aggregate patient data from seven clinical trials (n = 322) and eight registry studies (n = 1,090) underwent meta-analysis and pairwise analysis, revealing that eculizumab and ravulizumab had similar effects, including protective effects on TMAs and acute kidney injury, compared with the pre-/off-treatment state [31]. Comparatively, an advantage of our study over the previous study was the ability to balance prognostic baseline characteristics via access to patient-level data, rather than use only aggregate-level data. 

When interpreting the results of our indirect comparative efficacy analysis, we should note differences between treatment groups in characteristics for which it was not possible to balance using propensity scoring, which can only account for observed characteristics. These include differences in clinical practice associated with the involvement of different countries and with the trials being conducted at different times. For instance, the impact of the availability of an approved C5 inhibitor (eculizumab) at the time of the ravulizumab trial could not be accounted for, and no Asian centers participated in the eculizumab trials (treatment practice and a delay in treatment initiation has been suggested as a possible explanation for the lower cTMA response rate in the region [32]). Additionally, time to complement inhibitor treatment – a strong predictor of renal function recovery [33] – was inconsistently measured between studies, thus balancing could not be carried out on this parameter. Nonetheless, sensitivity and subgroup analyses supported the robustness and generalizability of the primary analysis findings, including when Asian centers were excluded from the analysis. While both eculizumab and ravulizumab have been shown to have a positive effect on patient outcomes, given its less frequent administration (7 infusions per year compared with 26 infusions in adults receiving eculizumab), ravulizumab has the potential to decrease treatment burden and disease management costs, as well as improve the overall quality of life for patients and caregivers owing to the reduced amount of time spent in treatment [34, 35]. 

The main limitation of this study relates to the small sample sizes available for analysis (39 eculizumab-treated patients and 46 ravulizumab-treated patients) due to the rarity of aHUS. This leads to a limited power to detect differences between treatments (unless large), as seen with the width of confidence intervals, which indicate a wide plausible range for the true differences on many comparisons made. Despite the small number of patients, the application of stabilized weights enabled the groups to be closely matched in baseline characteristics. 

In conclusion, in patients with aHUS, both eculizumab (dosed every 2 weeks) and ravulizumab (dosed less frequently, every 8 weeks) provide substantial improvements in a range of efficacy outcomes including renal function, hematological markers, and dialysis prevalence, as well as improvements in quality of life and measures of fatigue. In this small sample, using best practice methodology to adjust for observed differences in patient characteristics across clinical studies, no differences in outcomes were seen between ravulizumab and eculizumab after 26 weeks of treatment. 

## Acknowledgment 

Medical writing support was provided by Jessica Donaldson-Jones of Oxford PharmaGenesis Ltd, Oxford, UK and was funded by Alexion, AstraZeneca Rare Disease. The authors would like to thank Karl-Johan Myren and Yan Wang of Alexion, AstraZeneca Rare Disease, for their review of the first manuscript draft. 

## Funding 

This study was funded by Alexion, AstraZeneca Rare Disease. 

## Conflict of interest 

I. Tomazos, Å. Lommele, and P. Chen are employees of, and may own stocks/options in Alexion, AstraZeneca Rare Disease. A.J. Hatswell, K. Deighton, and E. Knowles are employees of Delta Hat Limited who were sponsored by Alexion, AstraZeneca Rare Disease to perform this analysis. S. Cataland has received research funding and consultancy fees from Alexion, AstraZeneca Rare Disease. N. Freemantle has received consultancy fees from Akcea, Alexion, AstraZeneca Rare Disease, Allergan, Alliance, AstraZeneca, Grifols, Ipsen, MSD, PTC, Sanofi, and Takeda. N.S. Sheerin has received consultancy fees from Alexion, AstraZeneca Rare Disease, AstraZeneca, Novartis, and Roche (all paid into departmental funds). E. Rondeau has received fees for expertise, consultancy, and scientific symposia from Alexion, AstraZeneca Rare Disease. 

**Table 1.** Definitions of dialysis status and outcome measures.

Dialysis status	Definition	
At baseline	Recorded as “yes” for patients who received dialysis within: • 5 days before study drug initiation (trials ALXN-aHUS-311 and ALXN-aHUS-312 (ravulizumab), and C10-003 and C10-004 (eculizumab)) • 7 days before study drug initiation (trial C08-002 (eculizumab))	
At endpoint	Recorded as “yes” for patients who received dialysis within: • 5 days before endpoint measure (trials ALXN-aHUS-311 and ALXN-aHUS-312 (ravulizumab)) • 7 days before endpoint measure (trials C08-002, C10-003, and C10-004 (eculizumab))	
Outcome measure	Improvement/normalization	Normal range
SCr concentration for non-dialysis patients	An improvement (decrease) in SCr of ≥ 25% from baseline sustained in ≥ 2 consecutive measures, ≥ 4 weeks apart Response to have been achieved at any time during the 26-week period	74 mmol/L ≤ observation ≤ 107 mmol/L
Platelet count	Platelet count ≥ 150 (×10^9^/L) sustained in ≥ 2 consecutive measures, ≥ 4 weeks apart Response to have been achieved at any time during the 26-week period	Observation ≥ 150 (×10^9^/L)
LDH concentration	LDH concentration < 246 U/L sustained in ≥ 2 consecutive measures, ≥ 4 weeks apart Response to have been achieved at any time during the 26-week period	Observation < 246 U/L
eGFR	An improvement of ≥ 15 mL/min/1.73m^2^ in eGFR, from baseline to 26 weeks	Observation ≥ 60 mL/min/1.73m^2^
FACIT-F subscale score	Improvement was derived using a threshold of a 3-point improvement from baseline to 26 weeks Higher scores indicate less fatigue	0 – 52 points
EQ-5D VAS score	Improvement was derived using a threshold of a 10-point improvement from baseline to 26 weeks Higher scores indicate better quality of life	
cTMA response	Criteria met simultaneously for SCr improvement, LDH normalization and platelet count normalization	See criteria for SCr improvement, LDH normalization, and platelet count normalization in the respective table rows above

cTMA = complete thrombotic microangiopathy; eGFR = estimated glomerular filtration rate; EQ-5D = 5-dimension EuroQol questionnaire; FACIT-F = Functional Assessment of Chronic Illness Therapy-Fatigue; LDH = lactate dehydrogenase; SCr = serum creatinine; VAS = visual analog scale.

**Equation 1 Equation1:**
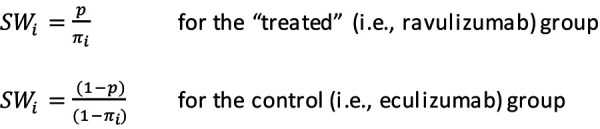
Equation 1

**Table 2.** Patient characteristics at baseline for adult native kidney patients, with application of stabilized weights.

Characteristic		Eculizumab n = 39	Ravulizumab n = 46	p-value for the difference between groups (95% CI)^a^
Patients by trial^b^, n (%)	ALXN-aHUS-311	0 (0)	46 (100)	
	C08-002	7.3 (19)	0 (0)	
	C10-004	31.7 (81)	0 (0)	
Sex, n (%)	Female	23.6 (61)	29.9 (65)	0.678 (–16%, 25%)
	Male	15.4 (39)	16.2 (35)	
Region, n (%)	Asia	0 (0)	10.4 (23)	0.002 (10%, 35%)
	Other world regions	39 (100)	35.6 (77)	
Dialysis at baseline, n (%)	Yes	20.5 (53)	24.2 (52)	0.998 (−21%, 21%)
	No	18.5 (47)	21.9 (48)	
Age, years	Mean (SD)	34 (13)	40 (14)	0.050 (0, 12)
Age, ≥ 65 years, n (%)	Yes	1.5 (4)	4.8 (10)	0.255 (−4%, 17%)
	No	37.4 (96)	41.2 (90)	
SCr concentration in non-dialysis patients, mmol/L	N	18	22	
	Mean (SD)	348 (231)	419 (301)	0.401 (−95, 238)
Platelet count, ×10^9^/L	Mean (SD)	118 (65)	118 (85)	0.979 (−32, 33)
LDH, U/L	Mean (SD)	534 (549)	664 (568)	0.285 (−111, 372)
eGFR, mL/min/1.73m^2^	Mean (SD)	16.6 (12.4)	16.7 (16.6)	0.996 (−6, 6)
eGFR category, mL/min/1.73m^2^, n (%)	≥ 90	0 (0)	0 (0)	
	60 – 89	0 (0)	3.3 (7)	
	45 – 59	2.4 (6)	1.3 (3)	
	30 – 44	4.1 (11)	1.8 (4)	
	15 – 29	5.2 (13)	5.5 (12)	
	< 15	27.2 (70)	34.2 (74)	
Systolic blood pressure, mmHg	N	39	43	
	Mean (SD)	143 (17)	145 (16)	0.457 (−5, 10)
FACIT-F subscale score	N	28	38	
	Mean (SD)	23 (14)	25 (15)	0.517 (−5, 9)
EQ-5D VAS	N	35	39	
	Mean (SD)	48 (18)	50 (30)	0.779 (−10, 13)
EQ-5D TTO	N	35	40	
	Mean (SD)	0.65 (0.31)	0.58 (0.34)	0.359 (−0.22, 0.08)

^a^Represents the 95% CI of the mean difference between treatments for continuous variables, and the 95% CI of the mean difference in proportions for categorical variables. For categorical variables, 95% CIs are presented only for binary outcomes and refer to the 95% CI around the difference between treatments for the first listed category (i.e., “Yes” for dialysis at baseline). ^b^Patients by trial before weighting: C08-002, n = 8; C10-004, n = 31. The application of stabilized weights did not change the effective sample size, calculated as the sum of weights in each treatment group. N is shown where patient data available differ from the overall number in each treatment group. Some values are given as decimal numbers owing to application of stabilized weights; n numbers represent outcome weights in each treatment group, the sum of which is the effective sample size. Percentages may not sum to 100% owing to rounding. CI = confidence interval; eGFR = estimated glomerular filtration rate; EQ-5D = 5-dimension EuroQol questionnaire; FACIT-F = Functional Assessment of Chronic Illness Therapy-Fatigue; LDH = lactate dehydrogenase; SCr = serum creatinine; SD = standard deviation; TTO = time trade-off; VAS = visual analog scale.

**Table 3.** Patient outcomes at 26 weeks for adult native kidney patients, with application of stabilized weights.

Outcomes		Eculizumab n = 39	Ravulizumab n = 46	p-value for the difference between groups (95% CI)^a^
Dialysis				
Yes	n (%)	3.1 (8)	9.7 (22)	0.070 (−1%, 30%)
	95% CI	3%, 21%	13%, 37%	
No	n (%)	35.9 (92)	33.3 (78)	
	95% CI	79%, 97%	63%, 87%	
Death				
Yes	n (%)	0 (0)	3 (7)	0.103 (−1%, 14%)
	95% CI	0%, 9%	2%, 18%	
No	n (%)	39 (100)	43 (93)	
	95% CI	91%, 100%	82%, 98%	
eGFR category, mL/min/1.73m^2^				
≥ 90	n (%)	2.7 (7)	12.3 (29)	
	95% CI	2%, 19%	17%, 44%	
60 – 89	n (%)	8 (20)	8.7 (20)	
	95% CI	11%, 35%	11%, 35%	
45 – 59	n (%)	8.1 (21)	1.9 (4)	
	95% CI	11%, 36%	1%, 15%	
30 – 44	n (%)	8.2 (21)	4 (9)	
	95% CI	11%, 36%	4%, 22%	
15 – 29	n (%)	5.6 (14)	3.6 (8)	
	95% CI	7%, 29%	3%, 20%	
< 15	n (%)	3.9 (10)	12.4 (29)	
	95% CI	4%, 23%	17%, 44%	
SCr concentration in non-dialysis patients, mmol/L				
N		36	33	
Mean (SD)		152 (75)	179 (281)	0.595 (−73, 127)
Platelet count, ×10^9^/L				
N		39	43	
Mean (SD)		244 (65)	243 (81)	0.953 (−33, 31)
LDH, U/L				
N		39	43	
Mean (SD)		179 (35)	200 (60)	0.059 (−1, 42)
eGFR, mL/min/1.73m^2^				
N		39	43	
Mean (SD)		51.4 (30.8)	55.4 (40.8)	0.619 (−12, 20)
Systolic blood pressure, mmHg				
N		39	43	
Mean (SD)		131 (16)	128 (19)	0.449 (−11, 5)
FACIT-F subscale score				
N		30	40	
Mean (SD)		40 (12)	43 (9)	0.382 (−3, 8)
EQ-5D VAS				
N		37	41	
Mean (SD)		74 (20)	79 (18)	0.260 (−4, 13)
EQ-5D TTO				
N		37	41	
Mean (SD)		0.89 (0.14)	0.89 (0.15)	0.890 (−0.06, 0.07)

^a^Represents the 95% CI of the mean difference between treatments for continuous variables, and the 95% CI of the mean difference in proportions for categorical variables. For categorical variables, 95% CIs are presented only for binary outcomes and refer to the 95% CI around the difference between treatments for the first listed category (i.e., “Yes” for dialysis at endpoint). N is shown where patient data available differ from the overall number in each treatment group. Some values are given as decimal numbers owing to application of stabilized weights; n numbers represent outcome weights in each treatment group, the sum of which is the effective sample size. Percentages may not sum to 100% owing to rounding. CI = confidence interval; CKD = chronic kidney disease; eGFR = estimated glomerular filtration rate; EQ-5D = 5-dimension EuroQol questionnaire; FACIT-F = Functional Assessment of Chronic Illness Therapy-Fatigue; LDH = lactate dehydrogenase; SCr = serum creatinine; TTO = time trade-off; VAS = visual analog scale.

**Table 4.** Patients achieving improvements/normalization at 26 weeks, with application of stabilized weights.

Outcomes		Eculizumab n = 39	Ravulizumab n = 46	p-value for the difference between groups (95% CI)^a^
Improvement from baseline in SCr concentration in non-dialysis patients				
N		17	18	
Yes	n (%)	13.8 (83)	13.5 (73)	0.509 (−36%, 18%)
	95% CI	59%, 94%	51%, 88%	
No	n (%)	2.9 (17)	4.9 (27)	
	95% CI	6%, 41%	12%, 49%	
Platelet count normalization				
N		39	43	
Yes	n (%)	37.6 (96)	39.5 (92)	0.391 (−14%, 6%)
	95% CI	85%, 99%	80%, 97%	
No	n (%)	1.4 (4)	3.5 (8)	
	95% CI	1%, 15%	3%, 20%	
LDH normalization				
N		39	43	
Yes	n (%)	36.9 (95)	38.3 (89)	0.372 (−17%, 6%)
	95% CI	83%, 98%	76%, 95%	
No	n (%)	2.1 (5)	4.7 (11)	
	95% CI	2%, 17%	5%, 24%	
Improvement from baseline in eGFR				
N		39	43	
Yes	n (%)	24.9 (64)	25.5 (59)	0.662 (−26%, 16%)
	95% CI	48%, 77%	44%, 73%	
No	n (%)	14.1 (36)	17.5 (41)	
	95% CI	23%, 52%	27%, 56%	
Improvement from baseline in FACIT-F subscale score				
N		28	37	
Yes	n (%)	25 (88)	30.8 (84)	0.623 (−21%, 13%)
	95% CI	72%, 96%	69%, 93%	
No	n (%)	3.3 (12)	5.9 (16)	
	95% CI	4%, 28%	7%, 31%	
Improvement from baseline in EQ-5D VAS				
N		35	38	
Yes	n (%)	30.4 (86)	31.4 (83)	0.687 (−20%, 13%)
	95% CI	71%, 94%	68%, 92%	
No	n (%)	4.8 (14)	6.4 (17)	
	95% CI	6%, 29%	8%, 32%	
Hematologic normalization				
N		39	43	
Yes	n (%)	35.5 (91)	35.8 (83)	0.294 (−22%, 7%)
	95% CI	78%, 97%	69%, 92%	
No	n (%)	3.5 (9)	7.2 (17)	
	95% CI	3%, 22%	8%, 31%	
cTMA response				
N		39	43	
Yes	n (%)	27.2 (70)	26.2 (61)	0.398 (−29%, 12%)
	95% CI	54%, 82%	46%, 74%	
No	n (%)	11.8 (30)	16.8 (39)	
	95% CI	18%, 46%	26%, 54%	
Time to cTMA response, days				
N		39	43	0.728 (−88, 62)
Mean (SD)		169 (167)	156 (174)	

^a^Represents the 95% CI of the mean difference between treatments for continuous variables, and the 95% CI of the mean difference in proportions for categorical variables. For categorical variables, 95% CIs are presented only for binary outcomes and refer to the 95% CI around the difference between treatments for the first listed category (i.e., “Yes” for cTMA response at endpoint). N is the number of patients with observations at both baseline and 26-weeks (both observations required for improvement/normalization endpoints). Some values are given as decimal numbers owing to application of stabilized weights; n numbers represent outcome weights in each treatment group, the sum of which is the effective sample size. Percentages may not sum to 100% owing to rounding. CI = confidence interval; cTMA = complete thrombotic microangiopathy; eGFR = estimated glomerular filtration rate; EQ-5D = 5-dimension EuroQol questionnaire; FACIT-F = Functional Assessment of Chronic Illness Therapy-Fatigue; LDH = lactate dehydrogenase; SCr = serum creatinine; VAS = visual analog scale.

## Supplemental material


