# Supplementary material for: Comparative efficacy of ravulizumab and eculizumab in the treatment of atypical hemolytic uremic syndrome: An indirect comparison using clinical trial data
Source: Clin Nephrol. 2021 Dec 21;97(5):261–72. doi: 10.5414/CN110516 (PMC9040196; doi:10.5414/CN110516)
Supplement: Supplementary file 1 [file clinnephrol-97-261-S01.pdf]

Supplementary Table 1. Patient outcomes at 26 weeks from the sensitivity analyses conducted for adult native kidney patients, after application of propensity score matching

		Eculizumab<br>N = 29	Ravulizumab<br>N = 29	P value for the difference between groups<br>(95% CI)[a]
**Dialysis at end point**				
Yes	n (%)	3 (10)	7 (26)	0.128<br>(−4%, 35%)
	95% CI	4%, 26%	13%, 45%	
No	n (%)	26 (90)	20 (74)	
	95% CI	74%, 96%	55%, 87%	
**Death**				
Yes	n (%)	0 (0)	2 (7)	0.150<br>(−2%, 16%)
	95% CI	0%, 12%	2%, 22%	
No	n (%)	29 (100)	27 (93)	
	95% CI	88%, 100%	78%, 98%	
**SCr concentration in non-dialysis patients, mmol/L**				
N		26	20	0.580<br>(−107, 186)
Mean (SD)		157 (75)	197 (308)	
**Platelet count, [$\times 10^9$/L]**				
N		29	27	0.758<br>(−48, 35)
Mean (SD)		238 (64)	232 (86)	
**LDH, U/L**				
N		29	27	0.175<br>(−9, 48)
Mean (SD)		184 (39)	204 (64)	
**eGFR, mL/min/1.73 m^2**				
N		29	27	0.519<br>(−13, 26)
Mean (SD)		46.5 (28.6)	52.7 (41.5)	
**Systolic blood pressure, mmHg**				
N		29	27	0.217<br>(−17, 4)
Mean (SD)		134 (17)	127 (21)	
**FACIT-F subscale score**				
N		24	25	0.316<br>(–3, 10)
Mean (SD)		39 (13)	42 (8)	
**EQ-5D VAS score**				
N		27	25	0.242<br>(−5, 18)
Mean (SD)		71 (21)	78 (20)	
**EQ-5D TTO**				
N		27	25	0.343<br>(−0.04, 0.12)
Mean (SD)		0.85 (0.15)	0.89 (0.14)	
**cTMA response**				
Yes	n (%)	19 (66)	16 (59)	0.629

	95% CI	47%, 80%	41%, 75%	(−32%, 19%)
No	n (%)	10 (34)	11 (41)	
	95% CI	20%, 53%	25%, 59%	
**Time to cTMA response, days**				
N		29	27	0.696 (−116, 78)
Mean (SD)		187 (175)	168 (185)	

[a]Represents the 95% CI of the mean difference between treatments for continuous variables, and the 95% CI of the mean difference in proportions for categorical variables. For categorical variables, 95% CIs are presented only for binary outcomes and refer to the 95% CI around the difference between treatments for the first listed category (i.e. 'Yes' for dialysis at end point).

N is shown where patient data available differ from the overall number in each treatment group. Percentages may not sum to 100% owing to rounding.

CI = confidence interval; cTMA = complete thrombotic microangiopathy; eGFR = estimated glomerular filtration rate; EQ-5D = 5-dimension EuroQol questionnaire; FACIT-F = functional assessment of chronic illness therapy-fatigue; LDH = lactate dehydrogenase; SCr = serum creatinine; TTO = time trade-off; VAS = visual analogue scale.

Supplementary Table 2. The number of patients eligible for each sensitivity analysis from each clinical trial

Non-transplant groups	Eculizumab		Ravulizumab
	C08-002	C10-004	ALXN-aHUS-311
Adults, no transplant	9	32	50
**Primary analysis**			
Adults, no transplant, complete cases for propensity score variables, and maximum of one missing laboratory measure	8	31	46
**Sensitivity analysis**			
Adults, no transplant, complete cases for propensity score variables, and maximum of one missing laboratory measure, outcome data within 28 days of the 6-month end point	7	31	45
Adults, no transplant, complete cases for propensity score variables, and maximum of one missing laboratory measure, excluding Asian countries	8	31	37
Adults, no transplant, complete cases for propensity score variables, and maximum of one missing laboratory measure, excluding deaths	8	31	43
Adults, no transplant, complete cases for propensity score variables, and maximum of one missing laboratory measure, excluding patients aged ≥ 65 years	7	30	41

Supplementary Table 3. Patient characteristics at baseline for adult patients with prior kidney transplant, with application of stabilized weights

Characteristic		Eculizumab N = 15	Ravulizumab N = 7	P value for the difference between groups (95% CI)[a]
**Effective sample size**		**N = 12.7**	**N = 9.3**	
**Patients by trial,[b] n (%)**	ALXN-aHUS-311	0 (0)	9.3 (100)	
	C08-002	4.6 (37)	0 (0)	
	C10-004	8 (63)	0 (0)	
**Sex, n (%)**	Female	9.4 (74)	1.6 (17)	0.009 (−91%, −22%)
	Male	3.3 (26)	7.7 (83)	
**Region, n (%)**	Asia	0 (0)	0.8 (8)	0.296 (−9%, 26%)
	Other world regions	12.7 (100)	8.5 (92)	
**Dialysis at baseline, n (%)**	Yes	3.3 (26)	1 (10)	0.361 (−46%, 15%)
	No	9.4 (74)	8.4 (90)	
**Age, years**	Mean (SD)	44 (15)	50 (10)	0.283 (−5, 17)
**Age, ≥ 65 years, n (%)**	Yes	0.8 (6)	1 (10)	0.730 (−20%, 28%)
	No	11.9 (94)	8.4 (90)	
**SCr concentration in non-dialysis patients, mmol/L**	N	9	8	
	Mean (SD)	336 (220)	358 (52)	0.760 (−123, 166)
**Platelet count, [$\times 10^9$/L]**	Mean (SD)	123 (49)	121 (44)	0.931 (−45, 42)
**LDH, U/L**	Mean (SD)	366 (127)	408 (81)	0.357 (−51, 136)
**eGFR, mL/min/1.73 m^2**	Mean (SD)	18 (11.1)	15.4 (2.6)	0.402 (−9, 4)
**Systolic blood pressure, mmHg**	N	12	9	
	Mean (SD)	148 (19)	133 (7)	0.017 (−27, −3)
**FACIT-F subscale score**	N	4	8	
	Mean (SD)	31 (14)	28 (12)	0.677 (−18, 12)
**EQ-5D VAS**	N	9	9	
	Mean (SD)	54 (22)	50 (18)	0.679 (−24, 16)
**EQ-5D TTO**	N	9	9	
	Mean (SD)	0.69 (0.21)	0.78 (0.18)	0.351 (−0.10, 0.28)

[a]Represents the 95% CI of the mean difference between treatments for continuous variables, and the 95% CI of the mean difference in proportions for categorical variables. For categorical variables, 95% CIs are presented only for binary outcomes and refer to the 95% CI around the difference between treatments for the first listed category (i.e. ‘Yes’ for dialysis at baseline).

[b]Patients by trial prior to weighting: ALXN-aHUS-311, n = 7; C08-002, n = 6; C10-004, n = 9.

N is shown where patient data available differ from the overall number in each treatment group. Some values are given as decimal numbers owing to application of stabilized weights; n numbers represent outcome weights in each treatment group, the sum of which is the effective sample size. Percentages may not sum to 100% owing to rounding.

CI = confidence interval; eGFR = estimated glomerular filtration rate; EQ-5D = 5-dimension EuroQol questionnaire; FACIT-F = functional assessment of chronic illness therapy-fatigue; LDH = lactate dehydrogenase; SCr = serum creatinine; SD = standard deviation; TTO = time trade-off; VAS = visual analogue scale.

Supplementary Table 4. Patient characteristics at baseline for pediatric native kidney patients, with application of stabilized weights

**Characteristic**		**Eculizumab N = 20**	**Ravulizumab N = 12**	**P value for the difference between groups (95% CI)[a]**
**Effective sample size**		**N = 21.3**	**N = 10.7**	
**Patients by trial, n (%)**	ALXN-aHUS-312	0 (0)	10.7 (100)	
	C08-002	0.9 (4)	0 (0)	
	C10-003	20.4 (96)	0 (0)	
**Sex, n (%)**	Female	9.9 (47)	7.1 (66)	0.291 (−16%, 55%)
	Male	11.4 (53)	3.6 (34)	
**Region, n (%)**	Asia	0 (0)	1.8 (17)	0.051 (−6%, 39%)
	Other world regions	21.3 (100)	8.9 (83)	
**Dialysis at baseline, n (%)**	Yes	7.7 (36)	3.9 (36)	0.995 (−35%, 35%)
	No	13.6 (64)	6.8 (64)	
**Age, years**	Mean (SD)	6 (5)	5 (3)	0.439 (−4, 2)
**SCr concentration in non-dialysis patients, mmol/L**	N	12	6	
	Mean (SD)	113 (38)	98 (53)	0.508 (−59, 29)
**Platelet count, [×10^9/L]**	Mean (SD)	75 (43)	64 (26)	0.373 (−36, 14)
**LDH, U/L**	Mean (SD)	2663 (2423)	2007 (1207)	0.317 (−1972, 660)
**eGFR, mL/min/1.73 m²**	Mean (SD)	32 (26.4)	31.8 (24.7)	0.984 (−19, 19)
**Systolic blood pressure, mmHg**	Mean (SD)	118 (12)	110 (16)	0.160 (−19, 3)
**FACIT-F subscale score**	N	13	6	
	Mean (SD)	26 (11)	27 (12)	0.754 (−10, 14)
**EQ-5D VAS**	N	NA	NA	
	Mean (SD)	NA	NA	NA
**EQ-5D TTO**	N	NA	NA	
	Mean (SD)	NA	NA	NA

[a]Represents the 95% CI of the mean difference between treatments for continuous variables, and the 95% CI of the mean difference in proportions for categorical variables. For

categorical variables, 95% CIs are presented only for binary outcomes and refer to the 95% CI around the difference between treatments for the first listed category (i.e. 'Yes' for dialysis at baseline).

[b]Patients by trial prior to weighting: ALXN-aHUS-312, n = 12; C08-002, n = 1; C10-003, n = 19.

N is shown where patient data available differ from the overall number in each treatment group. Some values are given as decimal numbers owing to application of stabilized weights; n numbers represent outcome weights in each treatment group, the sum of which is the effective sample size. Percentages may not sum to 100% owing to rounding.

CI = confidence interval; eGFR = estimated glomerular filtration rate; EQ-5D = 5-dimension EuroQol questionnaire; FACIT-F = functional assessment of chronic illness therapy-fatigue; LDH = lactate dehydrogenase; NA = insufficient data available; SCr = serum creatinine; SD = standard deviation; TTO = time trade-off; VAS = visual analogue scale.

Supplementary Table 5. Patient outcomes at 26 weeks for adult patients with prior kidney transplant, with application of stabilized weights

		Eculizumab N = 15	Ravulizumab N = 7	P value for the difference between groups (95% CI)[a]
**Effective sample size**		**N = 12.7**	**N = 9.3**	
**Dialysis at end point**				
Yes	n (%)	3 (24)	0.3 (4)	0.199 (−47%, 7%)
	95% CI	9%, 52%	0%, 36%	
No	n (%)	9.4 (76)	8.3 (96)	
	95% CI	48%, 91%	64%, 100%	
**Death**				
Yes	n (%)	0 (0)	0.4 (5)	0.430 (−9%, 19%)
	95% CI	0%, 23%	0%, 36%	
No	n (%)	12.7 (100)	8.9 (95)	
	95% CI	77%, 100%	64%, 100%	
**SCr in non-dialysis patients, mmol/L**				
N		8	8	0.189 (−26, 125)
Mean (SD)		185 (100)	235 (54)	
**Platelet count, [×10^9/L]**				
N		12	9	0.330 (−61, 21)
Mean (SD)		218 (65)	198 (27)	
**LDH, U/L**				
N		12	9	0.411 (−52, 22)
Mean (SD)		214 (57)	199 (26)	
**eGFR, mL/min/1.73 m^2**				
N		12	9	0.221 (−25, 6)
Mean (SD)		34.4 (25)	25.1 (9.3)	
**Systolic blood pressure, mmHg**				
N		12	9	< 0.001 (−39, −14)
Mean (SD)		140 (19)	113 (10)	
**FACIT-F subscale score**				
N		6	7	0.014 (3, 14)
Mean (SD)		38 (7)	47 (2)	
**EQ-5D VAS score**				
N		11	8	0.390 (−15, 6)
Mean (SD)		79 (12)	74 (10)	
**EQ-5D TTO**				
N		11	8	0.802 (−0.09, 0.11)
Mean (SD)		0.85 (0.14)	0.86 (0.07)	

**cTMA response**				
Yes	n (%)<br>95% CI	7.2 (59)<br>32%, 81%	7.1 (82)<br>49%, 96%	0.251<br>(−14%, 61%)
No	n (%)<br>95% CI	5.1 (41)<br>19%, 68%	1.6 (18)<br>4%, 51%	
**Time to cTMA response, days**				
N		12	9	0.171<br>(−250, 47)
Mean (SD)		235 (235)	134 (98)	

[a]Represents the 95% CI of the mean difference between treatments for continuous variables, and the 95% CI of the mean difference in proportions for categorical variables. For categorical variables, 95% CIs are presented only for binary outcomes and refer to the 95% CI around the difference between treatments for the first listed category (i.e. 'Yes' for dialysis at end point).

N is shown where patient data available differ from the overall number in each treatment group. Some values are given as decimal numbers owing to application of stabilized weights; n numbers represent outcome weights in each treatment group, the sum of which is the effective sample size. Percentages may not sum to 100% owing to rounding.

CI = confidence interval; cTMA = complete thrombotic microangiopathy; eGFR = estimated glomerular filtration rate; EQ-5D = 5-dimension EuroQol questionnaire; FACIT-F = functional assessment of chronic illness therapy-fatigue; LDH = lactate dehydrogenase; SCr = serum creatinine; TTO = time trade-off; VAS = visual analogue scale.

Supplementary Table 6. Patient outcomes at 26 weeks for pediatric native kidney patients, with application of stabilized weights

		Eculizumab N = 20	Ravulizumab N = 12	P value for the difference between groups (95% CI)[a]
**Effective sample size**		**N = 21.3**	**N = 10.7**	
**Dialysis at end point**				
Yes	n (%)	1.5 (7)	1.3 (12)	0.618 (−17%, 28%)
	95% CI	2%, 26%	3%, 42%	
No	n (%)	19.8 (93)	9.4 (88)	
	95% CI	74%, 98%	58%, 97%	
**Death**				
Yes	n (%)	0 (0)	0 (0)	1.000 (0%, 0%)
	95% CI	0%, 15%	0%, 26%	
No	n (%)	21.3 (100)	10.7 (100)	
	95% CI	85%, 100%	74%, 100%	
**SCr concentration in non-dialysis patients, mmol/L**				
N		20	9	0.734 (−17, 12)
Mean (SD)		40 (20)	38 (18)	
**Platelet count, [$\times 10^9$/L]**				
Mean (SD)		291 (93)	298 (72)	0.808 (−53, 67)
**LDH, U/L**				
Mean (SD)		456 (221)	256 (69)	0.001 (−309, −91)
**eGFR, mL/min/1.73 m^2**				
Mean (SD)		105.5 (39.6)	107.4 (59.3)	0.926 (−38, 41)
**Systolic blood pressure, mmHg**				
Mean (SD)		109 (11)	102 (10)	0.053 (−16, 0)
**FACIT-F subscale score**				
N		15	8	0.675 (−5, 3)
Mean (SD)		48 (6)	48 (5)	
**EQ-5D VAS score**				
N		NA	NA	NA
Mean (SD)		NA	NA	
**EQ-5D TTO**				
N		NA	NA	NA
Mean (SD)		NA	NA	

**cTMA response**				
Yes	n (%)<br>95% CI	14.3 (67)<br>46%, 83%	8.2 (77)<br>47%, 93%	0.565<br>(−22%, 42%)
No	n (%)<br>95% CI	7 (33)<br>17%, 54%	2.5 (23)<br>7%, 53%	
**Time to cTMA response, days**				
Mean (SD)		150 (135)	91 (117)	0.211<br>(−151, 34)

[a]Represents the 95% CI of the mean difference between treatments for continuous variables, and the 95% CI of the mean difference in proportions for categorical variables. For categorical variables, 95% CIs are presented only for binary outcomes and refer to the 95% CI around the difference between treatments for the first listed category (i.e. ‘Yes’ for dialysis at end point).

N is shown where patient data available differ from the overall number in each treatment group. Some values are given as decimal numbers owing to application of stabilized weights; n numbers represent outcome weights in each treatment group, the sum of which is the effective sample size. Percentages may not sum to 100% owing to rounding.

CI = confidence interval; cTMA = complete thrombotic microangiopathy; eGFR = estimated glomerular filtration rate; EQ-5D = 5-dimension EuroQol questionnaire; FACIT-F = functional assessment of chronic illness therapy-fatigue; LDH = lactate dehydrogenase; NA = insufficient data available; SCr = serum creatinine; TTO = time trade-off; VAS = visual analogue scale.

Plain language summary – comparative efficacy of ravulizumab and eculizumab in the treatment of atypical hemolytic uremic syndrome (aHUS): an indirect comparison using clinical trial data

Date of summary: August 2021

This is a summary of an analysis to compare how well ravulizumab and eculizumab worked as treatments for aHUS when studied in separate clinical trials.

- Ravulizumab and eculizumab are approved treatments for aHUS. However, as they have not been studied in a head-to-head clinical trial in aHUS, we cannot compare them directly. In cases like this, indirect treatment comparison methods can be used.

What is an indirect treatment comparison?

- Indirect treatment comparison is a valid and accepted method of comparing how well treatments work when the treatments had originally been studied in separate clinical trials.
- Comparing results between different clinical trials is challenging because there can be differences in the patient populations and in the ways of measuring how well the treatments work.
- The methods used balance the different trial populations to ensure a fair comparison.

What is aHUS?

- aHUS is a rare disease in which part of the immune system – the complement system – becomes overactive. This causes damage to the body, especially the kidneys.

What was done in this analysis?

- Changes in important disease features over a 26-week study period were compared for patients treated with either eculizumab 10 mg/mL or ravulizumab 10 mg/mL.
- Data were available from four clinical trials with eculizumab and two clinical trials with ravulizumab.

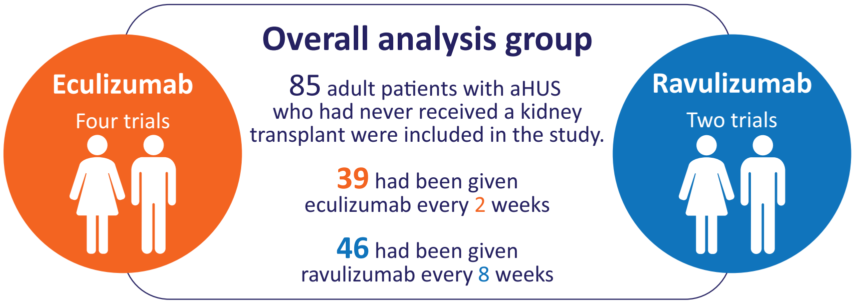

How were the treatment comparisons made?

- This study used a statistical technique called propensity score weighting to adjust for differences between patient populations (measured before treatment was given) from separate clinical trials and therefore balance the populations.
- The values included in the propensity score for balancing the populations were based on clinical expert input. The factors which needed to be accounted for were:
  - whether a patient is undergoing dialysis
  - estimated glomerular filtration rate (eGFR) – a measure of how well the kidneys are working
  - platelet count – a measure of how well the blood clots
  - serum lactate dehydrogenase (LDH) levels – a measure of aHUS disease activity
- The resulting scores were then used to equalize the characteristics between eculizumab and ravulizumab groups, meaning that disease features could be compared between the groups.

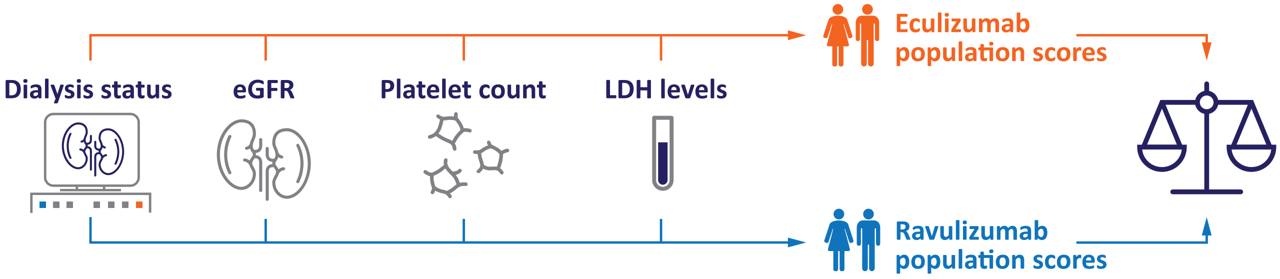

What did this study find?

- Compared with measures taken before treatments were given in the clinical trials, aHUS disease features were substantially improved after 26 weeks of treatment with eculizumab or with ravulizumab.
  - These data showed that the patient populations were well balanced for the comparison of treatments between groups.

		Measurements taken before treatments were given				Measurements taken after 26 weeks of treatment			
		Eculizumab 39 patients	Ravulizumab 46 patients	Treatment difference	95% confidence interval	Eculizumab 39 patients	Ravulizumab 46 patients	Treatment difference	95% confidence interval
**Kidneys:** fewer patients remained on dialysis, and kidney function improved suggesting the kidneys were working better.	Percentage of patients undergoing dialysis	53%	52%	1%	−21% to 21%	8%	22%	14%	−1% to 30%
	eGFR, mean, mL/min/1.73 m^2	16.6	16.7	0.1	−6 to 6	51.4	55.4	4	−12 to 20
**Blood:** the number of platelets in the blood increased; levels of serum LDH decreased, indicating reduced disease activity.	Platelet count, mean, x 10^9/L	118	118	0	−32 to 33	244	243	1	−33 to 31
	Serum LDH levels, mean, U/L	534	664	130	−111 to 372	179	200	21	−1 to 42
**Fatigue and quality of life:** patients had higher scores in questionnaires assessing disease impact on quality of life and levels of fatigue. This suggests that patients felt that their quality of life improved, and they felt less tired.	Quality of life questionnaire, mean score	48	50	2	−10 to 13	74	79	5	−4 to 13
	Fatigue questionnaire, mean score	23	25	2	−5 to 9	40	43	3	−3 to 8

- There was no statistical difference between eculizumab and ravulizumab groups (called treatment difference) for any of the disease features measured.
  - The researchers analyzed the data to account for the variability that often exists in clinical trial data. This was done by calculating confidence intervals for the treatment difference.

What are confidence intervals (CIs) and what do they mean?

- CI is a range of numbers that are higher or lower than a value; in this case, we are looking at treatment difference.
- A 95% CI means there is a 95% chance that the true treatment difference lies within the range.
- If 95% CIs for a value have a wide range, uncertainty is greater and the calculated value less precise, as there is a wider plausible range of values.
- Wide intervals might happen if there are small numbers of patients in a study.
- When analyzing the differences between eculizumab and ravulizumab groups, the 95% CIs were generally wide.
- If the 95% CIs for two groups being compared were narrow, such that there was no overlap between the two, it would be more likely that there is a statistically meaningful difference between the two groups.

Example:
Percentage of patients receiving dialysis

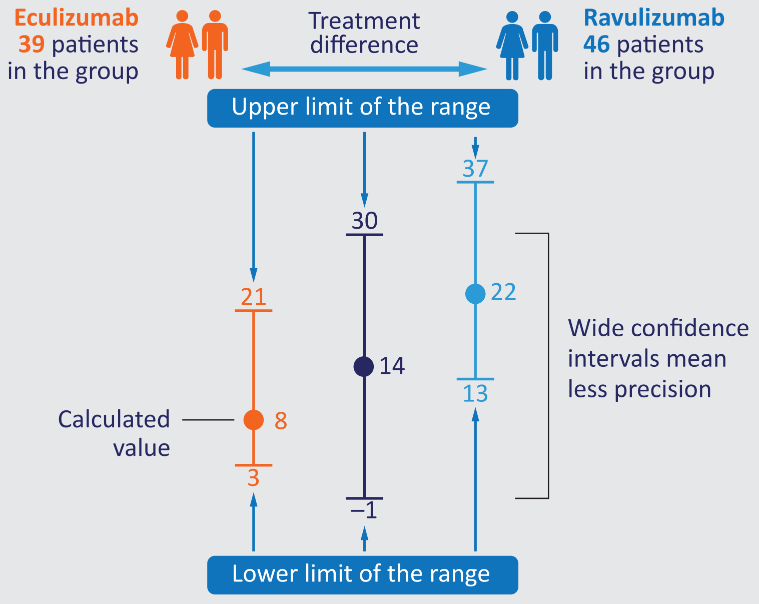

What are the limitations of a study like this?

- aHUS is a rare disease so a small number of patients were available for analysis.
- Having a small number of patients in the clinical trials means it is harder to detect differences between eculizumab and ravulizumab, especially if the differences we are looking for are small.

What were the main conclusions from this study?

- In patients with aHUS, both eculizumab (given every 2 weeks) and ravulizumab (given less frequently, every 8 weeks) improved a number of key disease features, suggesting both are effective at treating aHUS.
- No differences in disease features were seen between eculizumab- and ravulizumab-treated patients after 26 weeks of treatment, although the small number of patients included in the study meant that uncertainty was greater.

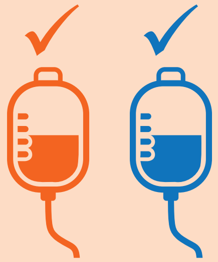

Who sponsored this analysis?

- Alexion, AstraZeneca Rare Disease, Boston, MA, USA.
- Alexion thanks all the patients and staff who participated in the clinical trials, as well as the independent patients and patient organizations for their reviews of this summary.

This summary is based on the following research article:

- Tomazos I *et al*. Comparative efficacy of ravulizumab and eculizumab in the treatment of atypical hemolytic uremic syndrome: an indirect comparison using clinical trial data. *Clinical Nephrology*; 2021.

Summary prepared by Jessica Donaldson-Jones, PhD, of Oxford PharmaGenesis. Funding for preparation of the summary and editorial review was provided by Alexion, AstraZeneca Rare Disease. Patient representatives for aHUS also provided editorial review. The original authors of the full article reviewed and approved the summary.
